# Metabolic syndrome is associated with worse prognosis in elderly patients with hepatocellular carcinoma

**DOI:** 10.3389/fonc.2025.1542328

**Published:** 2025-04-11

**Authors:** Junwei Huang, Yiming Tao, Jianguo Yao, Xiaorui Song, Yiming Li, Yiting Yuan

**Affiliations:** Department of General Surgery, The First People’s Hospital of Tongxiang, Tongxiang, Zhejiang, China

**Keywords:** metabolic syndrome, hepatocellular carcinoma, prognosis, elderly patients, survival analysis

## Abstract

**Background:**

Metabolic syndrome (MetS), a constellation of metabolic abnormalities such as obesity, hypertension, dyslipidemia, and insulin resistance, has been implicated in cancer progression. However, its impact on the prognosis of hepatocellular carcinoma (HCC) in elderly patients remains unclear. This study evaluates the relationship between MetS and survival outcomes in elderly patients undergoing hepatectomy for HCC.

**Methods:**

This retrospective cohort study enrolled elderly HCC patients (≥65 years) who underwent hepatectomy at The First People’s Hospital of Tongxiang between January 2018 and December 2022. Patients were categorized into MetS and non-MetS groups based on diagnostic criteria by the Chinese Diabetes Society. Propensity score matching (PSM) was performed, yielding 166 matched pairs. Overall survival (OS) and recurrence-free survival (RFS) were analyzed using Kaplan-Meier curves and Cox proportional hazards models, adjusted for potential confounding factors.

**Results:**

The 5-year recurrence (57.2% vs. 41.0%, *P* = 0.02) and mortality (33.1% vs. 17.5%, *P* < 0.01) rates were notably higher among patients with MetS compared to those without. Multivariate Cox regression showed that MetS was independently associated with a 1.43-fold increased risk of recurrence (95% CI: 1.02-2.00; *P* = 0.04) and a 1.73-fold increased risk of mortality (95% CI: 1.08–2.77; *P* = 0.02). A dose-response relationship was observed: each additional MetS component was associated with a 1.55-fold increased risk of recurrence (95% CI: 1.31–1.83; *P* < 0.01) and a 1.73-fold increased risk of mortality (95% CI: 1.39–2.17; *P* < 0.01).

**Conclusions:**

MetS is associated with significantly worse survival outcomes in elderly HCC patients, with mortality risk escalating as the number of MetS components increases.

## 1 Introduction

Hepatocellular carcinoma (HCC) ranks as the sixth most common cancer globally and the third leading cause of cancer-related mortality (1). Despite advances in therapeutic strategies such as surgical resection, liver transplantation, and systemic therapies, the prognosis for HCC remains dismal (2). This poor outcome is attributed to late-stage diagnosis in most patients and the complex interplay of factors influencing tumor progression and patient survival. Among these factors, host-related conditions such as metabolic comorbidities have emerged as potential contributors to HCC development and progression (3, 4).

Metabolic syndrome (MetS) is a cluster of interconnected metabolic abnormalities that includes central obesity, insulin resistance, hypertension, and dyslipidemia. Globally, the prevalence of MetS has increased markedly, paralleling the rise in sedentary lifestyles and obesity (5). Epidemiological studies have linked MetS to an increased risk of various cancers, including HCC, largely due to its association with chronic inflammation, insulin resistance, and altered lipid metabolism (6, 7). These mechanisms not only drive carcinogenesis but may also influence the biological behavior of established tumors, potentially impacting survival outcomes.

The relationship between MetS and HCC prognosis is of particular importance in elderly populations, as aging is accompanied by a higher prevalence of both MetS and HCC (8). In the elderly, comorbid conditions, polypharmacy, and diminished physiological reserves compound the challenges of cancer treatment, making the management of coexisting conditions, such as MetS, critical for improving outcomes. However, the prognostic significance of MetS in elderly patients with HCC remains poorly characterized. While studies have established the role of MetS in HCC risk (6, 9), its impact on survival outcomes is less clear, particularly in the context of elderly patients who may present with unique clinical and metabolic profiles.

This study aims to bridge these knowledge gaps by evaluating the association between MetS and survival outcomes in elderly patients with HCC. We hypothesize that MetS is associated with worse OS, with the risk increasing as the number of MetS components rises. By addressing these questions, this study seeks to provide evidence that could guide clinical decision-making and inform the design of interventions aimed at mitigating the impact of MetS on HCC prognosis.

## 2 Materials and methods

### 2.1 Patients

This retrospective cohort study was conducted at The First People’s Hospital of Tongxiang, focusing on elderly patients diagnosed with HCC between January 2018 and December 2022. The study adhered to the ethical guidelines of the Declaration of Helsinki and was approved by the Ethics Committee of Tongxiang First People’s Hospital (No. 2023043). Informed consent was waived by the Ethics Committee of Tongxiang First People’s Hospital due to the retrospective nature.

Inclusion criteria include, 1) age ≥65 years at the time of diagnosis; 2) histologically or radiologically newly diagnosed HCC in Barcelona Clinic Liver Cancer (BCLC) stage 0 and stage A; 3) underwent curative‐intent hepatectomy; 4) availability of complete clinical and follow-up data. Patients were excluded if they had coexistence of other primary malignancies or incomplete medical records. All patients underwent surgical treatment of a newly diagnosed HCC without preoperative treatments and the surgeries were performed by our experienced surgical team. After surgery, patients were followed up according to a standardized recurrence surveillance protocol.

### 2.2 Definition of MetS

The MetS is a cluster of metabolic abnormalities and there are several definitions. In this study, we adopted the criteria proposed by the Chinese Diabetes Society (10). The definition of MetS status requires the presence of any three or more of the following criteria: (1) obesity: body mass index ≥25 kg/m^2^ for Asians; (2) hyperglycemia: fasting blood glucose ≥6.1 mmol/L or 2-h plasma glucose ≥7.8 mmol/L or previously diagnosed; (3) hypertension: systolic/diastolic blood pressure ≥140/90 mm Hg or under antihypertensive therapy; (4) dyslipidemia: triglycerides ≥1.7 mmol/L or high-density lipoprotein cholesterol <0.9 mmol/L in men or <1.0 mmol/L in women. Patients were categorized into two groups based on the presence or absence of MetS at the time of HCC diagnosis.

### 2.3 Data collection and outcomes

Clinical data were extracted from electronic medical records, including demographic information, smoking history, American Society of Anesthesiologists (ASA) score, viral hepatitis status (HBV and HCV), surgical characteristics, histological characteristics, and MetS components. Cirrhosis was defined based on histopathological evidence of F4 fibrosis in nontumoral liver tissue, assessed using the Laennec cirrhosis scoring system, in accordance with established clinical and pathological guidelines (11, 12).

The primary endpoint was OS, defined as time from HCC diagnosis to death from any cause. Recurrence-free survival (RFS) was the secondary endpoint, and was estimated from HCC diagnosis until evidence of tumor relapse. All patients were followed up from initial admission after the surgery since January 2018 to death or last follow-up visit before December 2022, whichever occurred first. The data for patients who were alive at the time of the last follow-up or lost to follow-up before death were considered censored.

### 2.4 Statistical analysis

To minimize potential bias arising from baseline differences between the MetS and non-MetS groups, propensity score matching (PSM) was applied. Propensity scores were estimated through logistic regression, incorporating covariates such as age, sex, smoking status, ASA score and both surgical and histological characteristics. A 1:1 matching was conducted between MetS and non-MetS patients using a nearest-neighbor algorithm without replacement, with a caliper width set to 0.2 times the standard deviation of the logit of the propensity score.

Continuous variables were expressed as mean ± standard deviation (SD) or median with interquartile range (IQR), depending on the data distribution, and were compared using the Student’s t-test or Mann-Whitney U test. Categorical variables were presented as frequencies and percentages and analyzed using the chi-square test or Fisher’s exact test. Kaplan-Meier survival curves were constructed to compare OS and RFS between patients with and without MetS, with differences assessed using the log-rank test. Cox proportional hazards regression models were used to identify independent prognostic factors for OS and RFS. In the univariate analysis, the following variables were evaluated: age, sex, smoking status, ASA score, viral hepatitis status (HBV and HCV), surgical characteristics, histological characteristics, and MetS status. Variables with a *P* value <0.10 in the univariate analysis were sequentially included in the multivariate model using the forward selection method. Adjusted hazard ratios (HRs) and 95% confidence intervals (CIs) were reported for all significant variables in the final multivariate model.

All statistical analyses were performed using SPSS version 22.0, with a two-sided *P*<0.05 considered statistically significant.

## 3 Results

**Figure 1:**
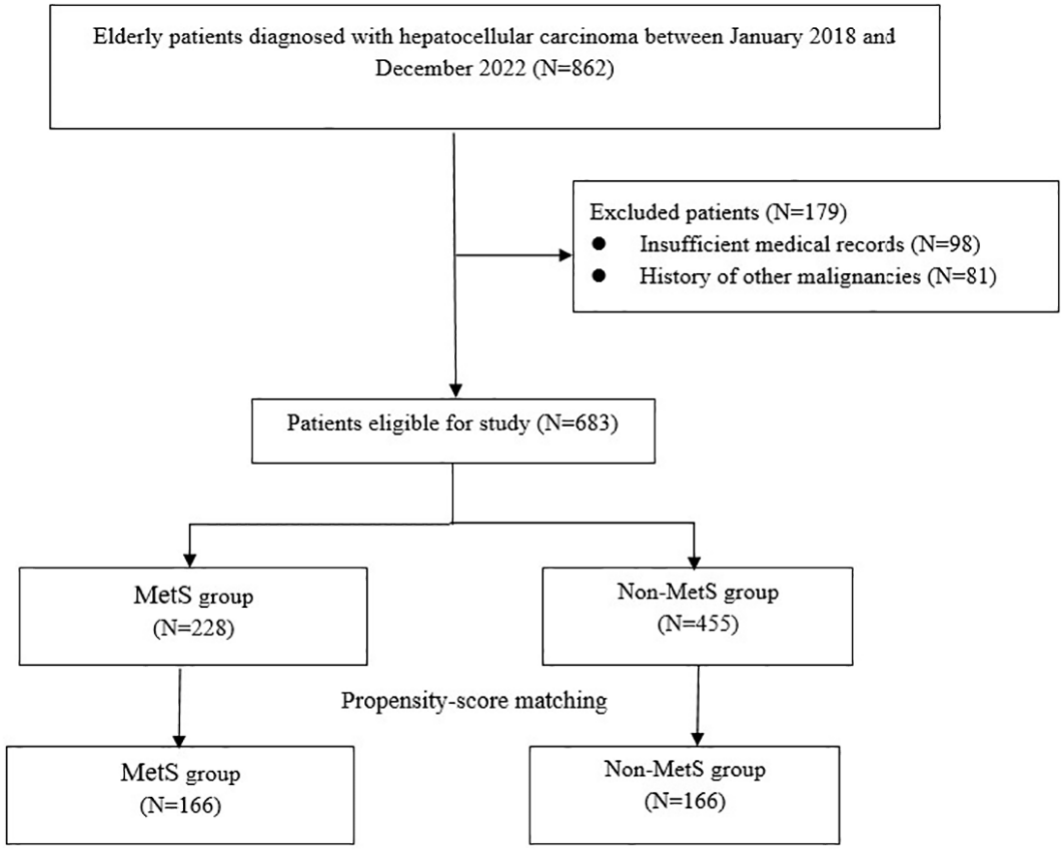
Patients’ selection flow.

A total of 862 patients with HCC were initially enrolled in this study. After excluding 179 cases, the final analysis included 683 participants, among whom 228 (33.4%) were identified as having MetS. PSM yielded 166 matched pairs, resulting in 332 patients for subsequent comparisons (**Figure 1**). As presented in **Table 1**, there were no significant differences in demographic factors such as age and sex, lifestyle factors like smoking, or clinical variables including surgical and histological characteristics between the MetS and non-MetS groups (P > 0.05). However, the MetS group exhibited significantly elevated levels of body mass index (BMI), systolic blood pressure (SBP), diastolic blood pressure (DBP), fasting glucose, triglycerides, and total cholesterol (all *P* < 0.01). Additionally, the prevalence of HBV and HCV infections was significantly higher in the MetS group (both *P*>0.05).

**Table 1:** Comparison of baseline characteristics between non-MetS and MetS groups.

	Non-MetS group (n = 166)	MetS group (n = 166)	*P* value
Age, years	69.0 ± 2.4	69.5 ± 2.4	0.96
Female	51 (30.7%)	43 (25.9%)	0.39
Current or ever smoker, n (%)	36 (21.7%)	35 (21.1%)	1.00
ASA score III to IV, n (%)	102 (61.4%)	97 (58.4%)	0.65
MetS number	1.4 ± 0.5	3.3 ± 0.4	<0.01
BMI, kg/m^2^	22.4 ± 2.6	25.8 ± 2.6	<0.01
SBP, mmHg	123.0 ± 11.5	142.7 ± 12.5	<0.01
DBP, mmHg	74.3 ± 11.1	86.2 ± 11.4	<0.01
Fasting glucose, mmol/L	5.6 ± 1.2	8.6 ± 1.5	<0.01
Triglyceride, mmol/L	1.1 ± 0.3	1.7 ± 0.4	<0.01
TC, mmol/L	4.4 ± 0.5	4.8 ± 0.6	<0.01
Viral hepatitis status, n (%)			
HBV infection	107 (64.5%)	139 (83.7%)	<0.01
HCV infection	10 (6.0%)	33 (19.9%)	<0.01
Surgical characteristics, n (%)			
Minimally invasive approach	69 (41.6%)	70 (42.2%)	1.00
Type of resection			
Limited resection	39 (23.5%)	53 (31.9%)	0.11
Segmentectomy	29 (17.5%)	21 (12.7%)	0.28
Sectionectomy	47 (28.3%)	56 (33.7%)	0.34
Hemi-hepatectomy	43 (25.9%)	53 (31.9%)	0.28
Major hepatectomy	10 (6.0%)	9 (5.4%)	1.00
Histological characteristics, n (%)			
Nontumoral liver fibrosis			0.14
F0 or F1	75 (45.2%)	62 (37.3%)	
F2	16 (9.6%)	30 (18.1%)	
F3	37 (22.3%)	35 (21.1%)	
F4	38 (22.9%)	39 (23.5%)	
Degree of steatosis			0.43
<5%	65 (39.2%)	60 (36.1%)	
5%-33%	67 (40.4%)	62 (37.3%)	
>33%	34 (20.5%)	44 (26.5%)	
Number of lesions			0.92
1	140 (84.3%)	140 (84.3%)	
2-3	23 (13.9%)	22 (13.3%)	
>3	3 (1.8%)	4 (2.4%)	
Size of lesions>5 cm	88 (53.0%)	86 (51.8%)	0.91
R0 resection	151 (91.0%)	158 (95.2%)	0.19
G3/G4 tumor grade	34 (20.5%)	26 (15.7%)	0.32
Macrovascular invasion	22 (13.3%)	23 (13.9%)	1.00
Microvascular invasion	55 (33.1%)	70 (42.2%)	0.11
Satellitosis	24 (14.5%)	36 (21.7%)	0.12

MetS, metabolic syndrome; ASA, American Society of Anesthesiologists; BMI, body mass index; SBP, systolic blood pressure; DBP, diastolic blood pressure; TC, total cholesterol; HBV, hepatitis B virus; HCV, hepatitis C virus.

Both recurrence (57.2% vs. 41.0%, *P* = 0.02) and mortality (33.1% vs. 17.5%, *P* < 0.01) rates were notably higher among patients with MetS compared to those without (**Table 2**). Further analysis of the causes of death revealed that tumor-related death was significantly more frequent in the MetS group (25.3% vs. 10.2%, *P* < 0.01), whereas no significant differences were observed in liver-related death, cardiopulmonary events, or other causes of death between the two groups (all *P* < 0.01). These findings suggest that the increased mortality in MetS patients is primarily driven by tumor progression rather than non-tumor-related complications.

**Table 2:** Comparison of recurrence and mortality between non-MetS and MetS groups.

	Non-MetS group (n = 166)	MetS group (n = 166)	P value
Recurrence during the follow-up	68 (41.0%)	91 (57.2%)	0.02
Death during the follow-up	29 (17.5%)	55 (33.1%)	<0.01
Cause of death			
Tumor-related death	17 (10.2%)	42 (25.3%)	<0.01
Liver-related death	4 (2.4%)	6 (3.6%)	0.75
Cardiopulmonary event	4 (2.4%)	4 (2.4%)	1.00
Other	4 (2.4%)	3 (1.8%)	1.00

**Figure 2:**
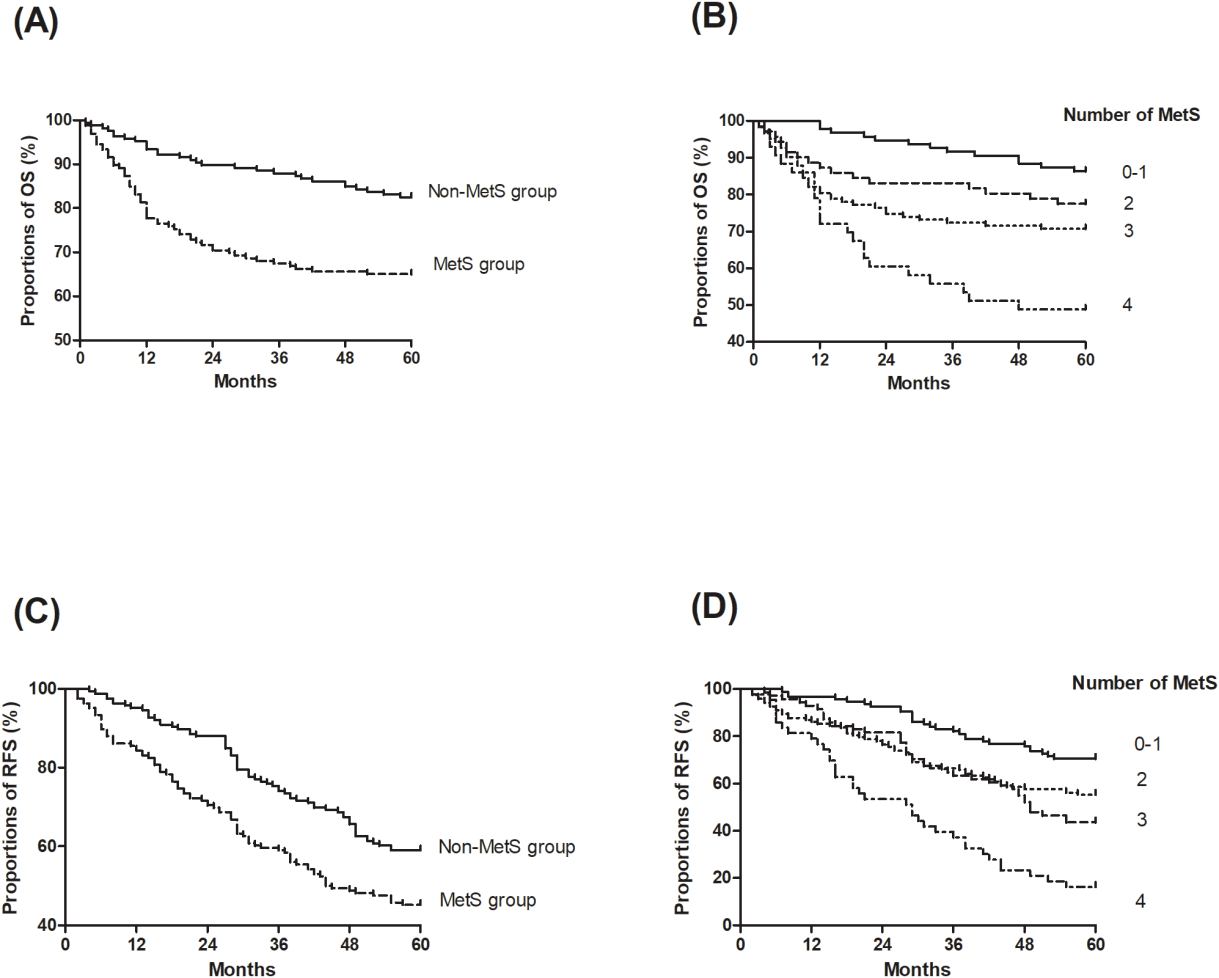
Kaplan-Meier curves of overall survival (OS) and recurrence-free survival (RFS) stratified by metabolic syndrome (MetS) status and the number of MetS components. **(A)** OS by MetS status; **(B)** OS by the number of MetS components; **(C)** RFS by MetS status; **(D)** RFS by the number of MetS components.

Kaplan-Meier survival analysis revealed a significant disadvantage in the MetS group, with the log-rank tests confirming poorer OS (**Figure 2A**. HR = 2.29, 95% CI: 1.50–3.50; *P* < 0.01) and RFS (**Figure 2C**. HR = 1.63, 95% CI: 1.19–2.24; *P* < 0.01). Multivariate Cox proportional hazards analysis, adjusted for variables including age, sex, smoking status, ASA score, viral hepatitis status, and surgical and histological features, demonstrated that MetS was independently associated with a 1.43-fold increased risk of recurrence (95% CI: 1.02-2.00; *P* = 0.04) and a 1.73-fold increased risk of mortality (95% CI: 1.08–2.77; *P* = 0.02) compared to the non-MetS group (**Table 3**). Additionally, other significant prognostic factors for mortality included cirrhosis (HR=4.99, 95% CI: 2.68–9.29; *P* < 0.01), HBV infection (HR=2.54, 95% CI: 1.29–5.04; *P* < 0.01), HCV infection (HR=2.21, 95% CI: 1.37–3.58; *P* < 0.01), and ASA score III to IV (HR=1.73, 95% CI: 1.04–2.91; *P* = 0.04). For recurrence, significant predictors included cirrhosis (HR=1.79, 95% CI: 1.21–2.64; *P* < 0.01), HBV infection (HR=1.59, 95% CI: 1.06–2.37; *P* = 0.03), and HCV infection (HR=2.19, 95% CI: 1.45–3.32; *P* < 0.01).

**Table 3:** Multivariate Cox regression for exploring the association between metabolic syndrome (MetS) with mortality and recurrence.

	Hazard ratios (HR)	95% confidence interval (CI)	P value
Factors associated with mortality			
MetS	1.73	1.08-2.77	0.02
ASA score III to IV	1.73	1.04-2.91	0.04
Cirrhosis	4.99	2.68-9.29	<0.01
HBV	2.54	1.29-5.04	<0.01
HCV	2.21	1.37-3.58	<0.01
Factors associated with recurrence			
MetS	1.43	1.02-2.00	0.04
Cirrhosis	1.79	1.21-2.64	<0.01
HBV	1.59	1.06-2.37	0.03
HCV	2.19	1.45-3.32	<0.01

MetS, metabolic syndrome; ASA, American Society of Anesthesiologists.

Age, sex, smoking status, viral hepatitis status, and surgical and histological features were adjusted for in the multivariate Cox analysis.

Additionally, a dose-response relationship was observed between the number of MetS components and survival outcomes (**Figures 2B, D**). Each additional MetS component was associated with a 1.55-fold increased risk of recurrence (95% CI: 1.31–1.83; *P* < 0.01) and a 1.73-fold increased risk of mortality (95% CI: 1.39–2.17; *P* < 0.01).

## 4 Discussion

### 4.1 Key findings

This study highlights MetS as a significant and independent predictor of poor OS in elderly patients with HCC. Patients with MetS demonstrated a 1.43-fold increased risk of recurrence and a 1.73-fold increased risk of mortality compared to those without, with the risk escalating as the number of MetS components increased. The dose-response relationship observed in this study emphasizes the cumulative impact of metabolic dysfunction on cancer prognosis, underscoring the need for integrated management strategies in this vulnerable population.

### 4.2 Comparison with previous studies

Our findings align with prior studies linking MetS to worse outcomes in various cancers, including HCC. For instance, Zhang et al. (13) evaluated the impact of MetS on the long-term prognosis of patients with hepatitis B virus-related HCC (HBV-HCC) after radical hepatectomy. Their study revealed that MetS was independently associated with poorer OS and RFS, with hazard ratios of 1.68 and 1.78, respectively. Similarly, a multicenter study of 1,753 patients with HCC reported that concurrent MetS was independently associated with a 30% increase in the risk of death, with poorer 5-year OS (47.5% vs. 61.0%) rates (14). Our findings expand on this by demonstrating the prognostic impact of MetS in an elderly cohort, a population often underrepresented in cancer research.

### 4.3 Mechanisms linking MetS to HCC outcomes

Elderly patients with HCC face distinct challenges, including diminished physiological reserves and higher prevalence of comorbidities, which compound the adverse effects of MetS (15, 16). Previous studies have demonstrated that individual components of MetS, such as diabetes and obesity, significantly worsen cancer outcomes. For instance, insulin resistance and hyperinsulinemia activate oncogenic pathways, including PI3K-Akt-mTOR, which promote tumor cell proliferation and angiogenesis (17). Dyslipidemia and altered lipid metabolism provide energy substrates, such as free fatty acids and cholesterol, that fuel tumor growth and metastasis (18). Chronic low-grade inflammation associated with MetS, driven by elevated levels of inflammatory cytokines (e.g., IL-6 and TNF-α), fosters a pro-tumorigenic microenvironment by activating signaling pathways like NF-κB and STAT3 (19). Additionally, obesity exacerbates oxidative stress, and hypertension contributes to vascular dysfunction and increased tumor neovascularization (20). MetS is also associated with impaired T-cell function and an increase in myeloid-derived suppressor cells (MDSCs), creating an immunosuppressive environment that facilitates immune evasion and tumor progression (21). These mechanisms collectively drive tumor progression and reduce the efficacy of oncologic treatments. Our findings extend this understanding by demonstrating a dose-response relationship, indicating that the cumulative burden of MetS components significantly amplifies the risk of mortality.

Moreover, our analysis of RFS revealed that MetS was independently associated with a 1.43-fold increased risk of recurrence, further underscoring the role of metabolic dysfunction in promoting tumor aggressiveness and recurrence. The dose-response relationship observed between the number of MetS components and recurrence risk suggests that MetS may directly influence tumor biology through mechanisms such as chronic inflammation, insulin resistance, and altered lipid metabolism. These findings highlight the importance of managing MetS components not only to improve overall survival but also to reduce the risk of tumor recurrence in HCC patients.

### 4.4 Limitations

This study has several limitations inherent to its retrospective design and the selection of an elderly cohort. First, selection bias may have occurred, as patients with more severe disease or comorbidities might have been more likely to have detailed medical records, potentially skewing the sample. Second, information bias, such as variability in the recording of MetS components or causes of death, could affect the accuracy of our findings. For example, the diagnostic criteria for MetS have evolved over time, and inconsistencies in its definition may have influenced patient classification. Third, despite our efforts to control for confounding variables through propensity score matching and multivariate analysis, unmeasured confounders such as socioeconomic status, lifestyle factors (e.g., diet, physical activity), and access to healthcare may have influenced the results. Additionally, the retrospective nature of this study precludes establishing causality; we can only report associations between MetS and HCC outcomes.

The heterogeneity of the elderly population further complicates the interpretation of our findings. Elderly patients often present with a wide range of health statuses and comorbidities, including frailty, cognitive impairment, and polypharmacy, which may independently affect both MetS and HCC outcomes. For instance, the severity of cirrhosis at the time of HCC diagnosis is a significant confounding factor that may influence both MetS and HCC prognosis. While we included cirrhosis as a covariate in our multivariate analysis, the Laennec scoring system used in this study may not fully capture the dynamic nature of liver dysfunction. Advanced cirrhosis can independently worsen prognosis and may interact with MetS components, such as insulin resistance and dyslipidemia, to exacerbate tumor progression. Furthermore, elderly patients who survive long enough to develop HCC may represent a select group with better overall health, potentially introducing survival bias. Treatment variability in elderly patients, such as differences in surgical candidacy or postoperative care, could also influence outcomes. While we attempted to control for these factors through propensity score matching and multivariate analysis, residual confounding may still exist.

### 4.5 Future directions

To address these limitations, future studies should incorporate direct comparative analyses between MetS-HCC and non-HCC MetS patients to clarify the relative contributions of cancer-specific and metabolic factors to mortality. Existing nationwide cohort studies, such as the US National Health and Nutrition Examination Survey (NHANES) (22) and the China Cardiometabolic Disease and Cancer Cohort (4C) Study (23), suggest that the mortality rate in non-HCC MetS patients is significantly lower than that observed in MetS-HCC patients, indicating that cancer-specific mechanisms (e.g., tumor progression and recurrence) likely drive the elevated mortality in MetS-HCC patients. Prospective designs are needed to assess dynamic changes in MetS components over time and evaluate the impact of interventions targeting metabolic parameters (e.g., weight management, glycemic control, and lipid regulation) on HCC outcomes. Additionally, more detailed assessments of liver function, such as MELD scores or transient elastography, should be included to better account for the impact of cirrhosis severity on HCC prognosis. Age-stratified analyses, including younger cohorts, are also essential to better understand the age-specific effects of MetS on HCC outcomes. Finally, investigations into the interplay between MetS and specific molecular pathways involved in HCC progression could provide valuable insights for the development of targeted therapies. These efforts would not only enhance our understanding of the complex relationship between metabolic health and cancer outcomes but also inform more effective, personalized treatment strategies for this high-risk population.

### 4.6 Conclusions

In conclusion, MetS significantly worsens the prognosis of elderly patients with HCC, with its impact amplified by the cumulative burden of metabolic abnormalities. Recognizing and managing MetS components—such as obesity, insulin resistance, and dyslipidemia—should be prioritized in the care of this population. Implementing targeted interventions, including weight management, glycemic control, and lipid regulation, may improve both metabolic health and oncologic outcomes. A holistic approach that integrates metabolic management with cancer treatment is essential to enhance survival and reduce recurrence risk in elderly HCC patients with MetS.

## Data Availability

The raw data supporting the conclusions of this article will be made available by the authors, without undue reservation.

## References

[1] Samant H Amiri HS Zibari GB. Addressing the worldwide hepatocellular carcinoma: epidemiology, prevention and management. J Gastrointest Oncol. (2021) 12:S361–S73. doi: 10.21037/jgo.2020.02.08 PMC8343080 34422400

[2] Gao YX Ning QQ Yang PX Guan YY Liu PX Liu ML. Recent advances in recurrent hepatocellular carcinoma therapy. World J Hepatol. (2023) 15:460–76. doi: 10.4254/wjh.v15.i4.460 PMC10190692 37206651

[3] Campbell C Wang T McNaughton AL Barnes E Matthews PC. Risk factors for the development of hepatocellular carcinoma (Hcc) in chronic hepatitis B virus (Hbv) infection: A systematic review and meta-analysis. J Viral Hepat. (2021) 28:493–507. doi: 10.1111/jvh.13452 33305479 PMC8581992

[4] Ma Y Wang J Xiao W Fan X. A review of masld-related hepatocellular carcinoma: progress in pathogenesis, early detection, and therapeutic interventions. Front Med (Lausanne). (2024) 11:1410668. doi: 10.3389/fmed.2024.1410668 38895182 PMC11184143

[5] Saklayen MG. The global epidemic of the metabolic syndrome. Curr Hypertens Rep. (2018) 20:12. doi: 10.1007/s11906-018-0812-z 29480368 PMC5866840

[6] Jinjuvadia R Patel S Liangpunsakul S. The association between metabolic syndrome and hepatocellular carcinoma: systemic review and meta-analysis. J Clin Gastroenterol. (2014) 48:172–7. doi: 10.1097/MCG.0b013e3182a030c4 PMC3887366 24402120

[7] Yau STY Leung E Wong MCS Hung CT Chong KC Lee A. Metabolic dysfunction-associated profiles and subsequent site-specific risk of obesity-related cancers among chinese patients with diabetes: A retrospective cohort study. BMJ Open. (2024) 14:e082414. doi: 10.1136/bmjopen-2023-082414 PMC11146369 38569684

[8] Zheng Z Hu Y Ren Y Mo G Wan H. Correlation between metastatic patterns and age in patients with metastatic primary liver cancer: A population-based study. PloS One. (2023) 18:e0267809. doi: 10.1371/journal.pone.0267809 36706100 PMC9882911

[9] Cho Y Cho EJ Yoo JJ Chang Y Chung GE Choi IY. The importance of metabolic syndrome status for the risk of non-viral hepatocellular carcinoma: A nationwide population-based study. Front Oncol. (2022) 12:863352. doi: 10.3389/fonc.2022.863352 35600376 PMC9116136

[10] Hu D Peng F Lin X Chen G Zhang H Liang B. Preoperative metabolic syndrome is predictive of significant gastric cancer mortality after gastrectomy: the fujian prospective investigation of cancer (Fiesta) study. EBioMedicine. (2017) 15:73–80. doi: 10.1016/j.ebiom.2016.12.004 27979733 PMC5233804

[11] Xu XY Ding HG Li WG Xu JH Han Y Jia JD. Chinese guidelines on the management of liver cirrhosis (Abbreviated version). World J Gastroenterol. (2020) 26:7088–103. doi: 10.3748/wjg.v26.i45.7088 PMC7723671 33362370

[12] Bedossa P Poynard T. An algorithm for the grading of activity in chronic hepatitis C. The metavir cooperative study group. Hepatology. (1996) 24:289–93. doi: 10.1002/hep.510240201 8690394

[13] Zhang KJ Ye TW Lu WF Xu FQ Xie YM Wang DD. Impact of metabolic syndrome on the long-term prognosis of patients with hepatitis B virus-related hepatocellular carcinoma after hepatectomy. Front Oncol. (2022) 12:1042869. doi: 10.3389/fonc.2022.1042869 36338761 PMC9632286

[14] Wang MD Tang SC Li C Sun LY Xu X Liang YJ. Association of concurrent metabolic syndrome with long-term oncological prognosis following liver resection for hepatocellular carcinoma among patients with chronic hepatitis B virus infection: A multicenter study of 1753 patients. Ann Surg Oncol. (2023) 30:346–58. doi: 10.1245/s10434-022-12529-6 36114441

[15] Brunot A Le Sourd S Pracht M Edeline J. Hepatocellular carcinoma in elderly patients: challenges and solutions. J Hepatocell Carcinoma. (2016) 3:9–18. doi: 10.2147/JHC.S101448 27574587 PMC4994800

[16] Cho E Cho HA Jun CH Kim HJ Cho SB Choi SK. A review of hepatocellular carcinoma in elderly patients focused on management and outcomes. In Vivo. (2019) 33:1411–20. doi: 10.21873/invivo.11618 PMC6755010 31471386

[17] Olatunde A Nigam M Singh RK Panwar AS Lasisi A Alhumaydhi FA. Cancer and diabetes: the interlinking metabolic pathways and repurposing actions of antidiabetic drugs. Cancer Cell Int. (2021) 21:499. doi: 10.1186/s12935-021-02202-5 34535145 PMC8447515

[18] Jin HR Wang J Wang ZJ Xi MJ Xia BH Deng K. Lipid metabolic reprogramming in tumor microenvironment: from mechanisms to therapeutics. J Hematol Oncol. (2023) 16:103. doi: 10.1186/s13045-023-01498-2 37700339 PMC10498649

[19] Siegel AB Zhu AX. Metabolic syndrome and hepatocellular carcinoma: two growing epidemics with a potential link. Cancer. (2009) 115:5651–61. doi: 10.1002/cncr.24687 PMC3397779 19834957

[20] Fosam A Perry RJ. Current mechanisms in obesity and tumor progression. Curr Opin Clin Nutr Metab Care. (2020) 23:395–403. doi: 10.1097/MCO.0000000000000690 32868685 PMC10059279

[21] Chen Z Han F Du Y Shi H Zhou W. Hypoxic microenvironment in cancer: molecular mechanisms and therapeutic interventions. Signal Transduct Target Ther. (2023) 8:70. doi: 10.1038/s41392-023-01332-8 36797231 PMC9935926

[22] Li Z Yang X Yang J Yang Z Wang S Sun F. The cohort study on prediction of incidence of all-cause mortality by metabolic syndrome. PloS One. (2016) 11:e0154990. doi: 10.1371/journal.pone.0154990 27195697 PMC4873211

[23] Lu J Li M He J Xu Y Zheng R Zheng J. Association of social determinants, lifestyle, and metabolic factors with mortality in chinese adults: A nationwide 10-year prospective cohort study. Cell Rep Med. (2024) 5:101656. doi: 10.1016/j.xcrm.2024.101656 39067445 PMC11384959

